# A prognostic nomogram integrating novel biomarkers identified by machine learning for cervical squamous cell carcinoma

**DOI:** 10.1186/s12967-020-02387-9

**Published:** 2020-06-05

**Authors:** Yimin Li, Shun Lu, Mei Lan, Xinhao Peng, Zijian Zhang, Jinyi Lang

**Affiliations:** 1 School of Medicine, University of Electronic Science and Technology of China, No. 2006, Xiyuan Avenue, High-tech Zone (West District), Chengdu, 611731 Sichuan People’s Republic of China; 2 Department of Radiation Oncology, Sichuan Cancer Hospital & Institute, Sichuan Cancer Center, School of Medicine, University of Electronic Science and Technology of China, No. 55, South Renmin Avenue Fourth Section, Chengdu, 610041 Sichuan People’s Republic of China; 3 Radiation Oncology Key Laboratory of Sichuan Province, No. 55, South Renmin Avenue Fourth Section, Chengdu, 610041 Sichuan People’s Republic of China; 4 Department of Oncology, Xiangya Hospital Central South University, Kaifu District, Changsha, 410008 Hunan People’s Republic of China

**Keywords:** Cervical squamous cell cancer, Weighted gene co-expression network analysis, Least absolute shrinkage and selection operator, Prognostic biomarkers, Nomogram

## Abstract

**Background:**

Cervical cancer (CC) represents the fourth most frequently diagnosed malignancy affecting women all over the world. However, effective prognostic biomarkers are still limited for accurately identifying high-risk patients. Here, we provided a combination machine learning algorithm-based signature to predict the prognosis of cervical squamous cell carcinoma (CSCC).

**Methods and materials:**

After utilizing RNA sequencing (RNA-seq) data from 36 formalin-fixed and paraffin-embedded (FFPE) samples, the most significant modules were highlighted by the weighted gene co-expression network analysis (WGCNA). A candidate genes-based prognostic classifier was constructed by the least absolute shrinkage and selection operator (LASSO) and then validated in an independent validation set. Finally, based on the multivariate analysis, a nomogram including the FIGO stage, therapy outcome, and risk score level was built to predict progression-free survival (PFS) probability.

**Results:**

A mRNA-based signature was developed to classify patients into high- and low-risk groups with significantly different PFS and overall survival (OS) rate (training set: p < 0.001 for PFS, p = 0.016 for OS; validation set: p = 0.002 for PFS, p = 0.028 for OS). The prognostic classifier was an independent and powerful prognostic biomarker for PFS in both cohorts (training set: hazard ratio [HR] = 0.13, 95% CI 0.05–0.33, p < 0.001; validation set: HR = 0.02, 95% CI 0.01–0.04, p < 0.001). A nomogram that integrated the independent prognostic factors was constructed for clinical application. The calibration curve showed that the nomogram was able to predict 1-, 3-, and 5-year PFS accurately, and it performed well in the external validation cohorts (concordance index: 0.828 and 0.864, respectively).

**Conclusion:**

The mRNA-based biomarker is a powerful and independent prognostic factor. Furthermore, the nomogram comprising our prognostic classifier is a promising predictor in identifying the progression risk of CSCC patients.

## Background

Cervical cancer (CC) represents the fourth most frequently diagnosed malignancy and the fourth leading cause of cancer-related death among females in 2018 worldwide [1]. Currently, the early diagnosis rate of cervical cancer has been improved after the introduction of cytologic screening and high-risk human papillomavirus (HPV) DNA testing, while the incidence has been decreased due to the development of vaccines against HPV. Comprehensive treatment, including the combination of bevacizumab, has achieved a favorable outcome for patients with cervical cancer [2–4]. However, 15–61% of women with stage I–III will experience metastatic disease, usually within the first 2 years of completing treatment [5]. Furthermore, for women with disease progression, the median overall survival ranges from 7 to 53 months [6]. So it appears that cervical cancer with similar baseline features is comprised of different groups with distinct outcomes. This heterogeneity within cervical cancer may be attributed to differences in molecular characterization. Currently, the International Federation of Gynecology and Obstetrics (FIGO) stage, lymph node status and clinicopathological features of the primary tumor are the most important prognostic variables for cervical cancer [7, 8], but these traditional prognostic factors do not help predict which patient will suffer disease progression.

With the rapid development of genomic sequencing technology, there has been increasing interest in the identification of molecules that are intimately associated with tumor phenotype and clinical behavior. In pursuit of molecules with better predictive value for cervical cancer, previous investigations have reported valuable biomarkers such as COX-2 [9, 10], p53 [11], VEGF [12], and Ki‑67 [13]. Recently, more candidate molecules have been identified [14–16]. However, the prognostic relevance of some biological factors requires further investigation because of a lack of high throughput data or failure of validation from independent centers. Although several biomarkers have been applied to predict the clinical outcome of patients with cervical cancer, their sensitivity and/or specificity remain unsatisfactory. Therefore, it is extremely urgent to identify more valuable biomarkers for diagnosing and monitoring recurrence and evaluating prognosis [17, 18].

In the present study, a combination machine learning algorithm-based strategy was developed to build robust prognostic models by using the RNA sequencing (RNA-seq) data from our retrospective cervical squamous cell carcinoma (CSCC) patient cohort. External RNA-seq datasets about CSCC with clinical follow-up details were carefully reviewed in Gene Expression Omnibus (GEO), The Cancer Genome Atlas (TCGA), and Oncomine databases. The eligible dataset was used as an independent validation set for the prognostic value. The performance of the Cox regression verified that our classifier was independent of clinical features. Furthermore, a nomogram was generated to predict the 1-, 3- and 5-year progression-free survival in the training cohort and evaluated in the independent validation cohort.

## Methods and materials

### Patients and clinical data

A total of 36 CSCC patients who underwent concurrent radiochemotherapy in Sichuan Cancer Hospital between 2013 and 2018 were included in the training set, based on the following criteria: (1) histologically confirmed CSCC; (2) availability of adequate archival formalin-fixed paraffin-embedded (FFPE) tissue collected prior to treatment; and (3) availability of complete clinical and follow-up data. Clinical staging was performed or updated according to the FIGO staging of cancer of the cervix uteri (2018) [19] and the 8th edition of the International Union Against Cancer (UICC)/American Joint Committee on Cancer (AJCC) Tumor Node Metastasis (TNM) classification [20] (Additional file 1: Table S1).

TCGA is a database comprised of high throughput genetic information of different cancer types. To avoid the batch effect from different platforms, the Genomic Data Commons (GDC) Legacy Archive (https://portal.gdc.cancer.gov/legacy-archive) was chosen to acquire the raw gene counts data and corresponding clinical information of human cervical carcinoma [21]. The selection criterion of the data for external validation was as follows: experimental strategy (RNA-Seq), data category (Gene expression), data type (Gene expression quantification), platform (Illumina HiSeq), workflow type (HTSeq—Counts), and clinicopathological information (detailed FIGO/TNM stage, therapy outcome, and survival information). Finally, the validation dataset contains 252 CSCC tissues.

The median follow-up time was 36 months for the training set, and 22.5 months for the validation set, respectively. The progression-free survival (PFS) was calculated from the date of initial diagnosis until progression or death, whichever came first, or last follow-up examination. The overall survival (OS) was estimated from the date of initial diagnosis to death or last follow-up examination. The protocol was approved by the ethics committee of Sichuan Cancer Hospital and carried out according to the principles of the Declaration of Helsinki. Informed consent was obtained from all patients in the training cohort for the acquisition and use of tissue samples and clinical data.

### Clinical samples and RNA sequencing

The 36 FFPE samples were obtained from patients in the training cohort. Total RNA was extracted from archived FFPE specimens with the RNeasy FFPE Kit (Qiagen GmbH, Hilden, Germany) after deparaffinization with Xylene. Paired-end libraries were synthesized from 100 ng/ml of total RNA using SMARTer Stranded Total RNA-Seq Kit v2 (Takara Bio, Japan) according to the manufacturer’s instructions. Sequence data were obtained using the Illumina NovaSeq 6000 platform.

### Weighted gene co-expression network analysis

The weighted gene co-expression network analysis (WGCNA), which was first proposed in 2005 [22], is based on the concept that genes in the same module share biological functions and/or are controlled by a common mechanism [23]. A batch of highly co-expressed genes is grouped into modules based on similarities in expression profiles among samples, and different modules are involved in individual functions [24]. WGCNA is increasingly being used to identify candidate biomarkers or therapeutic targets [25, 26].

The “variance stabilizing transformation (VST)” function from “DESeq 2” was used to obtain a normalized gene expression matrix. Then gene coexpression network analysis and hub genes screening were performed by the “WGCNA” package. The procedure was as follows: (1) Outlier samples were removed to ensure that the results of network construction were reliable. (2) A soft threshold (power = 4) was selected by standard scale-free model fitting index R^2^ = 0.703. (3) The adjacency matrix, a measurement of topology similarity, was transformed into a topological overlap matrix (TOM), and the corresponding dissimilarity (1-TOM) was calculated. (4) The hierarchical clustering dendrogram was plotted with identified modules, which were composed of a cluster of interconnected genes. (5) The module eigengenes (MEs) were calculated to evaluate the correlation between the modules and the clinical traits. (6) The hub genes in the significant modules (4 modules in the present study) were extracted. (7) The correlation coefficients between the gene significance (GS) with module membership (MM) were calculated and the p values were obtained.

### Least absolute shrinkage and selection operator

The least absolute shrinkage and selection operator (LASSO) is a machine learning algorithm in which both variable selection and regularization occur simultaneously. This penalized regression uses the L1 penalty which equals to the absolute value of the magnitude of coefficients to limit the size of the coefficients and then yields models with few coefficients (sparse models), and some coefficients can become zero and eliminate. Therefore, this model uses a penalty to shrink regression coefficients toward zero, a number of variables will be eliminated because their coefficients will shrink to exactly zero. This technique is quite suitable for analyzing gene expression profile, which is high dimensionality and small sample size [27, 28].

According to WGCNA results, a batch of genes in modules that were closely related to the prognosis of human CSCC was obtained. Those genes were used to identify the most powerful prognostic markers. In the present study, the LASSO regression model was performed with the package “glmnet” and the penalty parameter “lambda” was selected to choose the best model based on leave-one-out cross-validation, which is more suitable than tenfold cross-validation for a smaller number of samples [29, 30]. Finally, we extracted variables with nonzero coefficients and their corresponding coefficients. Combining coefficients with the relative expression levels of the selected RNAs (RNA_i_), a risk score for each patient was calculated: ${\text{Risk score}} = \mathop \sum \limits_{i = 1}^{n} RNA_{i} \times Coe_{i}$, in this formula the Coe_i_ represents the coefficient of each mRNA from the model.

### Statistical analysis and graphics

All statistical analyses and graphics were performed by using R software (R version 3.5.2). The associations of clinical characteristics between the training set and validation set were examined by Chi square test or Fisher’s exact test. The distributions of selected genes between groups were estimated and tested by the Wilcoxon rank-sum test. The differential expression genes (DEGs) were calculated using Bioconductor packages of “DESeq 2”. The “pheatmap” package was used for heat maps. The optimal cutoff value, sensitivity, specificity, and Youden index were calculated via time-dependent ROC curves by using the “survivalROC” package. The concordance index (C-index) of the risk scores was computed in the “survcomp” package and compared by the Student t-test. Kaplan–Meier curves and log-rank tests were employed to analyze PFS and OS rates in the “survival” package. The Cox proportional hazards regression model was also performed in the package “survival”. The package “forestplot” was used for the presentation of the results of the univariable and multivariable analysis. The nomogram was formulated and validated by using the “rms” package. All statistical tests were two-sided.

## Results

### Cohort characteristics

**Fig. 1:**
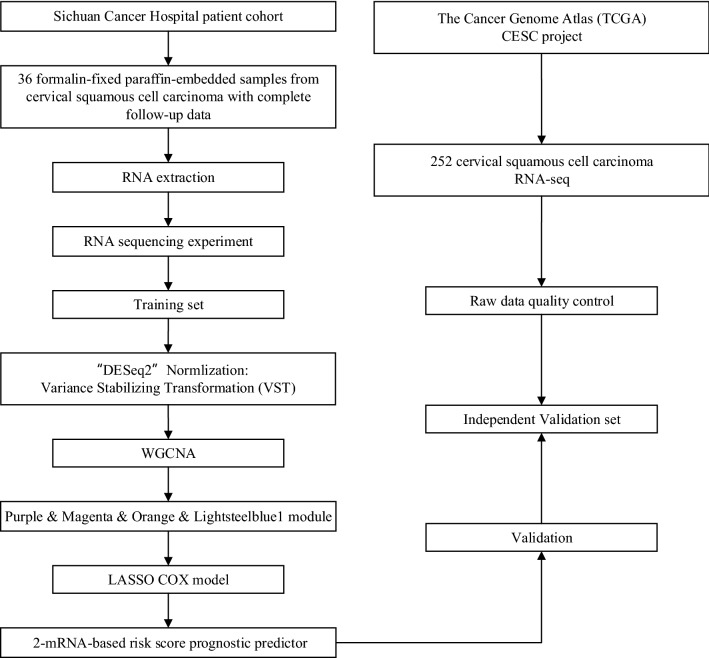
Flowchart of the prognostic model construction process

The study design and workflow are indicated in Fig. 1. A total of 288 patients with CSCC from two independent datasets were recruited, the baseline characteristics of these patients were summarized in Additional file 1: Table S2. In the training set, there was no patient within FIGO stage I, T0-1 or M1 CSCC, and all 36 patients were treated with concurrent radiochemotherapy. In contrast, there were 125 patients within the FIGO stage I CSCC from the validation set, which were mainly treated with hysterectomy in the TCGA-CESC project. The other significantly different characteristic between two datasets was smoking status, 3% of patients in the training set had smoked for more than 3 years and 21% in the validation set (p = 0.018).

### Weighted gene co-expression networks

One sample was deleted as an outlier after the hierarchical clustering analysis (Additional file 2: Figure S1A). Then a co-expression network was constructed using 35 cervical squamous cancer samples with complete clinical data (Additional file 2: Figure S1B). By the selected power of β = 4 (scale-free R^2^ = 0.703) as the soft-thresholding (Additional file 2: Figure S2A), a total of 46 modules were identified (Additional file 2: Figure S2B). The highest association in the module-trait relationship was found between 4 modules (purple, magenta, orange, and lightsteelblue1) and vital status (p < 0.01) (Additional file 2: Figure S3). Next, the gene significance was calculated to quantify the associations of individual genes in 4 modules with vital status. For each module, the MM was used to quantitatively measure the correlation of the selected module and the gene expression profile. Scatterplots in Additional file 2: Figure S4 showed significantly positive correlations of module membership with gene significance in vital status. As a result, the 1360 RNAs in 4 modules closely related to the prognosis of human CSCC were considered as candidates for identifying prognostic markers in our cohort.

### Construction of prognostic classifier by LASSO

**Fig. 2:**
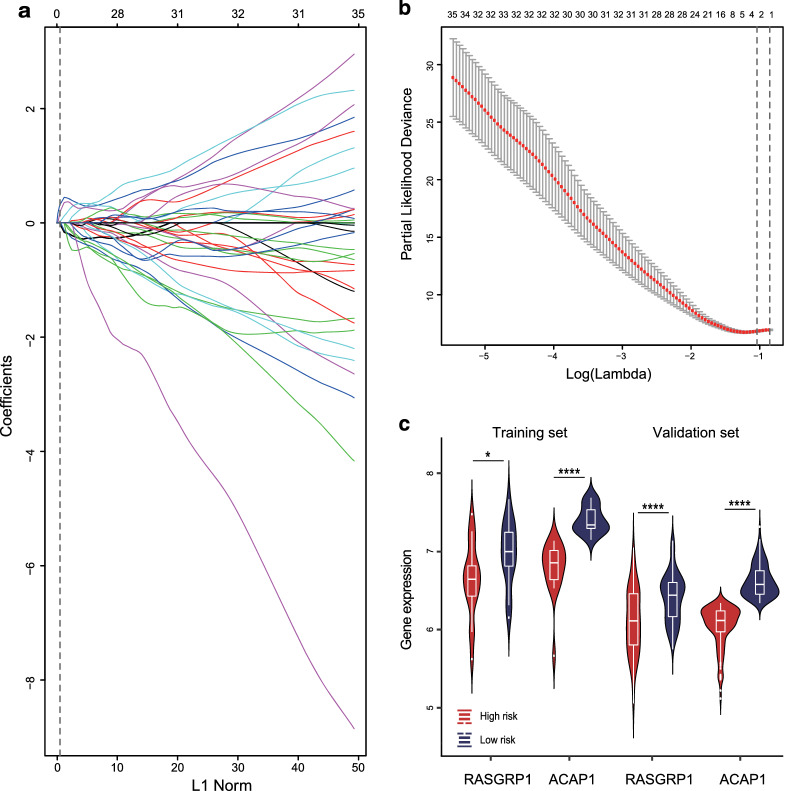
Construction of the prognostic model based on the risk score. **a** LASSO coefficient profiles of the 2 survival-related mRNAs. Each curve corresponds to a gene. It shows the path of its coefficient against the L1-norm of the whole coefficient vector at various λ values. The vertical line is drawn at the value λ = 0.2828604 chosen by leave-one-out cross-validation. Two genes (ACAP1 and RASGRP1) intersecting with the vertical line were chosen to build the final model. **b** Partial likelihood deviance for the LASSO coefficient profiles. The red dotted line stands for the cross-validation curve, error bars represent the upper and lower standard deviation curves along the λ sequence. The left vertical line shows the optimal λ value at which the minimum mean squared error is achieved and the corresponding genes. The right vertical line is for the most regularized model whose mean squared error is within 1 standard error of the minimal. It is indicated that the genes identified by optimal λ are the simplest model with the best performance. In **a**, **b**, the axis above indicates the number of genes involved in the LASSO model. **c** The expression levels of ACAP1 and RASGRP1 between low and high-risk groups in the training and validation set. The high-risk group (risk score ≥ 0.6715958) had significantly lower proportions of ACAP1 and RASGRP1 than the low-risk group (risk score < 0.6715958) in both datasets. All p values were corrected by the Bonferroni method. (Wilcoxon rank-sum test, *p-value < 0.05, ****p-value < 0.0001)

In the LASSO Cox regression model of the training set, a sequence of models was returned by the function “glmnet”. The optimal penalty parameter (λ = 0.2828604) was chosen by leave-one-out cross-validation via minimum criteria. We obtained 2 variables (ACAP1 and RASGRP1) with nonzero coefficients (Fig. 2a, b). Patients with higher ACAP1 showed significantly longer OS and PFS in both the training set (Additional file 2: Figure S5A, B) and validation set (Additional file 2: Figure S6A, B) (p < 0.05), and RASGRP1 demonstrated the same prognostic value (p < 0.05) except for PFS in the training set (p = 0.09) (Additional file 2: Figures S5C, D, S6C, D).

A risk score was calculated based on the 2 mRNAs’ expression status and model coefficients for each sample in the training set: risk score = (0.29457828* status of ACAP1) + (0.08243926* status of RASGRP1). A score of 0.6715958 was determined as the optimal cutoff value with the maximum Youden index to separate patients into a low-risk group (risk score < 0.6715958) and high-risk group (risk score ≥ 0.6715958). In the validation set, risk scores were then calculated for each patient and the same cutoff value was used to divide patients into different groups. When comparing the expression levels of 2 genes between groups, we found that the high-risk CSCC group had significantly lower proportions of ACAP1 and RASGRP1 than the low-risk group in both the training and validation set (p < 0.05) (Fig. 2c). When a genome-wide differential gene expression analysis (DEA) was performed between high- and low-risk groups, ACAP1 and RASGRP1 were found to be significantly lower in high-risk groups, but fold change did not reach the threshold of differential expression (Additional file 2: Figure S7A). Additionally, when DEA was performed between tumor and normal tissues from the validation set, it could be seen that both genes were highly expressed in tumor tissues, but RASGRP1 was a differentially expressed gene, while ACAP1 was not (Additional file 2: Figure S7B).

### Validation of the risk score predictor for prognosis

**Fig. 3:**
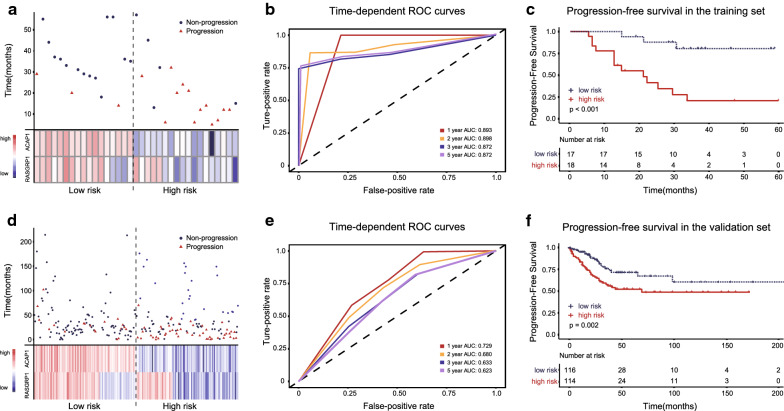
The disease progression status and gene expression levels of each patient, time‐dependent receiver operating characteristic (ROC) curve, and survival analysis based on the prognostic classifier in the training set and validation set. **a** The distribution of disease progression status (upper panel) and gene expression levels (lower panel) of each patient between low and high-risk groups in the training set. Each point in the scatterplots represents a patient, and the shape of the point represents whether the disease has progressed or not. The corresponding heatmap below each point vertically represents the expression level of ACAP1 and RASGRP1 genes in this patient. The patients were ordered according to the risk score level. **b** Time-dependent ROC curves in the training set. The area under the curves (AUC) at 1-, 2-, 3-, and 5-year were used to evaluate the prognostic accuracy. **c** Survival analysis of the different risk groups in the training set according to the optimal cutoff value (log‐rank test p‐value < 0.001). **d** The distribution of disease progression status (upper panel) and gene expression levels (lower panel) of each patient between low and high-risk groups in the validation set. The patients were ordered according to the risk score level. **e** Time-dependent ROC curves in the validation set. **f** Kaplan–Meier curves show the distinct outcome between low‐ and high‐risk groups in the validation set (log‐rank test p‐value = 0.002)

As shown in Fig. 3a, d, two scatterplots and heatmaps were used to investigate the relationships between disease progression status and expression levels of selected genes in the training and validation set, respectively. Each point in the scatterplots represents a patient, and the shape of the point represents whether the disease has progressed or not. The corresponding heatmap below each point vertically represents the expression level of ACAP1 and RASGRP1 genes in this patient. It can be seen that the expression patterns of ACAP1 and RASGRP1 in both datasets were quite similar. Patients from the low-risk group tended to express a higher ACAP1 and RASGRP1 level, whereas patients from the high-risk group incline to express a lower level.

**Fig. 4:**
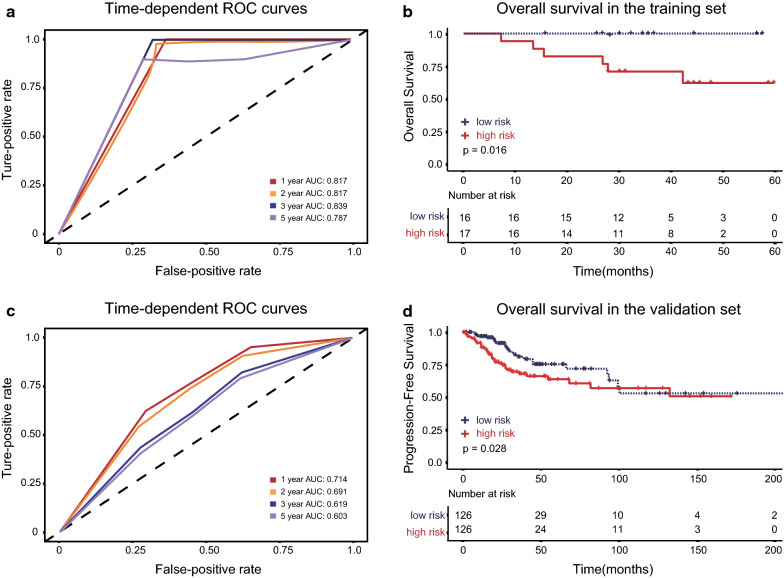
The time‐dependent ROC curve, and overall survival based on the prognostic classifier in the training set and validation set. **a** In the training set, the time-dependent ROC curve analysis showed the area under the curve (AUC) for OS at 1-, 2-, 3- and 5-year was 0.817, 0.817, 0.839 and 0.787, respectively, **b** high-risk score significantly predicted poor OS (log‐rank test p = 0.016). **c** In the validation set, the AUC for OS at 1-, 2-, 3- and 5-year was 0.714, 0.691, 0.619 and 0.603, respectively, **d** high risk score significantly predicted poor OS (log‐rank test p = 0.028)

In the training set, the time-dependent ROC curve analysis showed the area under the curve (AUC) for PFS at 1-, 2-, 3- and 5-year was 0.893, 0.898, 0.872 and 0.872, respectively (Fig. 3b), while the AUC for OS at 1-, 2-, 3- and 5-year was 0.817, 0.817, 0.839 and 0.787, respectively (Fig. 4a). We next sought to investigate the prognostic value of the risk score using the Kaplan–Meier survival curves and the log-rank test. The 1-, 2-, 3- and 5-year PFS rate of low-risk group was 100%, 87.8% (95% CI 73.4–100), 80.5% (95% CI 62.8–100) and 80.5% (95% CI 62.8–100) respectively, whereas it was only 61.1% (95% CI 42.3–88.3), 34.4% (95% CI 17.4–68.1), 20.6% (95% CI 7.7–55.5), and 20.6% (95% CI 7.7–55.5) for the high-risk group (p < 0.001, Fig. 3c). Similarly, the OS rate was significantly lower in the high-risk group compared with the low-risk group (p = 0.016) (Fig. 4b).

The 2-gene-based risk score prognostic predictor was then tested in the validation set. The AUC showed the ability of our model in predicting PFS (Fig. 3e) and OS (Fig. 4c) at 1-, 2-, 3- and 5-year. Then by Kaplan–Meier analysis and log-rank test, we found that the 1-, 2-, 3- and 5-year PFS rate of low-risk group in the validation set was 94.5% (95% CI 90.3–98.9), 87.4% (95% CI 80.9–94.4), 75.6% (95% CI 66.2–86.4) and 71.4% (95% CI 61.1–83.4) respectively, while it was 79.9% (95% CI 72.8–87.8), 66.5% (95% CI 57.9–76.3), 58.2% (95% CI 48.9–69.3), and 52.8% (95% CI 42.9–64.9) for the high-risk group (p = 0.002, Fig. 3f). Likewise, patients with higher risk scores had a significantly lower OS rate than their low-risk counterparts (p = 0.028). The median OS of the high-risk and low-risk group was 103 months(95% CI 56—176 months) and 136 months (95% CI 56—200 months), respectively (Fig. 4d).

### Cox proportional hazards regression model

**Fig. 5:**
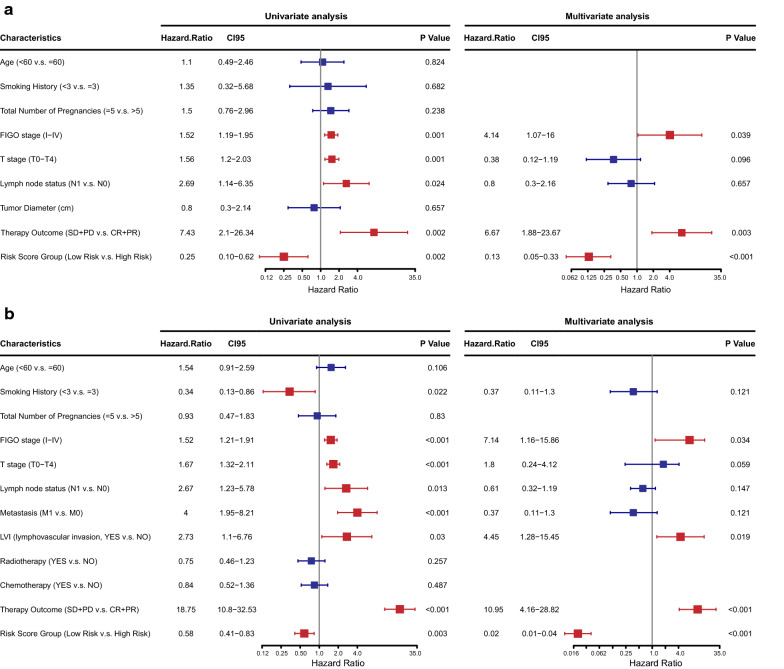
Forest plot of hazard ratios for PFS assessed by the prognostic classifier and clinicopathological characteristics in the **a** training set and **b** validation set. Error bars represent 95% confidence intervals

To verify whether the risk score classifier is independent of other clinicopathologic features, the effect on PFS was analyzed by Cox proportional hazards regression in the training and validation set. As shown in Fig. 5, in addition to FIGO stage, lymphovascular invasion (LVI) and therapy outcome, which are already well-known risk factors, multivariate analyses demonstrated that lower risk score remained a powerful and independent factor for a better PFS (training set: hazard ratio [HR] = 0.13, 95% CI 0.05–0.33, p < 0.001; validation set: HR = 0.02, 95% CI 0.01–0.04, p < 0.001).

Furthermore, the predictive performance of the risk score classifier was compared with a 9-gene signature [31] and a 10-gene signature [32], respectively. According to the studies, the 9-gene signature was established to predict recurrence and the 10-gene signature was for OS. The risk score of both signatures was calculated according to the coefficients provided by the primary studies. The C-indices were then computed to assess the predictive power of different models in the validation set. The 2-mRNA based signature had a significantly higher C-index in predicting PFS (p‐value = 0.023) (Additional file 2: Figure S8A) and OS (p‐value = 0.001) (Additional file 2: Figure S8B) at each follow-up duration.

### Construction and calibration of the nomogram for PFS

**Fig. 6:**
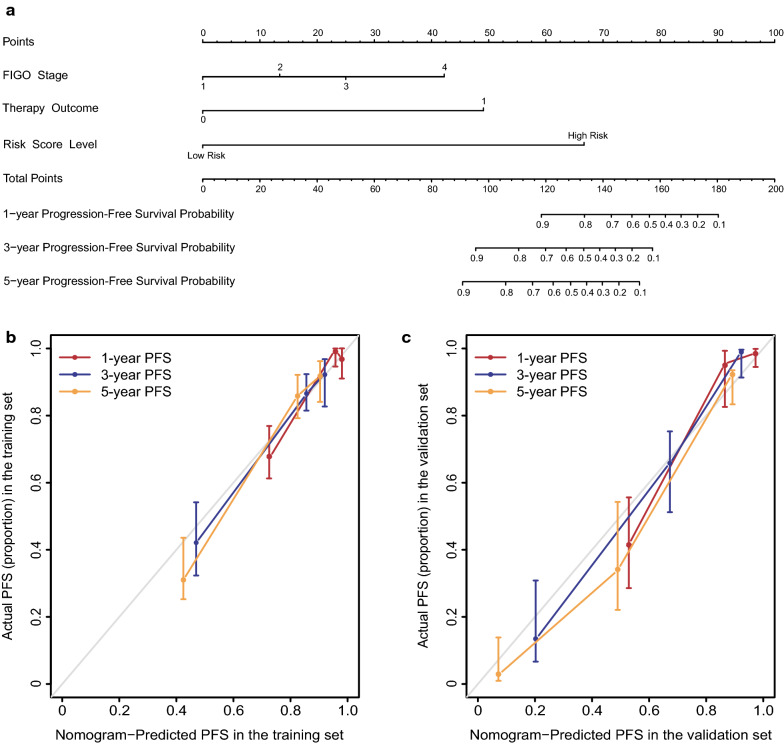
Prognostic nomogram for PFS at 1-, 3- and 5-year and the calibration curves in both cohorts. **a** The nomogram was developed in the training cohort with the risk score level, Figo stage, and therapy outcome incorporated. Depending on the value which is located on the 3 variable axes for each patient, a score will be assigned by drawing a line from the value on each variable axis up to the “points” scale. By summing all of the scores and locating it on the “total points” scale, we were easily able to draw a vertical line down to the PFS probability axis to determine the likelihood of 1-, 3- and 5- year PFS. The calibration curves for predicting PFS at each time point in the **b** training set and **c** validations set. Nomogram-predicted PFS is plotted on the x-axis; actual PFS is plotted on the y-axis

Nomograms are the visualization of statistical predictive models specifically developed to provide a more individualized prediction of outcome based on a combination of characteristics of each patient. Based on the results of the multivariable analysis, a nomogram comprising the independent prognostic factors was formulated to predict the 1-, 3-, and 5-year PFS in the training cohort (Fig. 6a). The risk score level that divided patients into low risk and the high-risk group was found to have the largest contribution to prognosis, followed by therapy outcome and FIGO stage. Each category within the 3 variables was assigned a score on the “points” scale at the top. By summing all of the scores and locating it on the “total points” scale, we were easily able to draw a vertical line down to the PFS probability axis. Then the estimated probability of 1-, 3- and 5- year PFS was determined. The calibration plots showed that the bias-corrected line of 1-, 3- and 5-year PFS were close to the ideal curve, which indicated a good agreement between predicted and actual PFS in both the training and external validation cohort (Fig. 6b, c). The C-index of the nomogram was 0.828 (95% CI 0.728–0.927) in the training cohort, 0.864 (95% CI 0.791–0.938) in the external validation cohort.

## Discussion

In this study, the sequencing data of the training cohort was obtained by RNA-seq experiments in FFPE tumor tissues from Chinese patients with CSCC. The 4 modules with a significantly positive relation to vital status were identified by WGCNA, and the selection was narrowed to 2 candidate mRNAs by LASSO Cox regression. Subsequently, according to the optimal cut-off point of risk score identified by the ROC analysis, CSCC patients in the training set were divided into low- and high-risk groups whose PFS and OS were significantly different. The reliability of our prognostic classifier was further confirmed in the independent validation set, indicating excellent reproducibility. The expression patterns of 2 mRNAs between low and high-risk groups in both RNA-seq based datasets were quite similar. More importantly, the results of the Cox proportional hazards regression model indicated that our classifier had a similar prognostic ability to the FIGO stage and therapy outcome, and could act as an independent factor for CSCC prognosis in both cohorts. Finally, based on the multivariate analysis of PFS, we built a nomogram including the FIGO stage, therapy outcome, and risk score level to predict PFS probability. The performance of the nomogram was verified in the validation cohorts from TCGA. The C-index (0.864) and highly fitted calibration plots revealed that our nomogram could provide simple and accurate prognosis predictions for 1-, 3- and 5-year PFS of CSCC.

WGCNA is a widely used approach to identify hub genes correlated with clinical traits in the data mining process. However, previous studies constructed the co-expression network mainly based on the filtering genes by differential expression analysis (DEA) [33, 34], which can lead to losing some potential genes and invalidate the scale-free topology assumption [35]. Recently, several studies have reported on the transcriptional profiles of cervical cancer. The first study identified a series of markers by performing the LASSO Cox regression model [31]. Three other studies selected and validated the prognostic signatures in a single dataset [32, 36, 37]. However, these studies used only one algorithm to select markers or lacked independent validation samples. To overcome these issues, a combination strategy from two distinct machine learning algorithms was developed based on the data without DEA to minimize the possibility of ignoring important biomarkers. Then the candidates were validated in an independent cohort.

Interestingly, both mRNAs in our model were found novelly to be associated with CSCC. Functions of ACAP1 in mediating endocytic recycling [38, 39] and cell migration [40] have been investigated already, but there is limited information on human cancers. The protein product of ACAP1, a GTPase-activating protein (GAP), activates the ADP-ribosylation factor 6 (ARF6) [41]. The amounts of ACAP1 are higher in highly invasive breast cancer cell lines than in weakly invasive or noninvasive cell lines [42]. As a key transport effector in the recycling of integrin β1, the inhibition of ACAP1 activation would lead to the suppression of glioma cell invasion [43]. These results suggest the potential involvement of ACAP1 in cancer progression. On the other hand, the role of RASGRP1 in tumorigenesis and progression remains controversial. Zhang et al. found that RASGRP1 was upregulated in hepatocellular carcinoma (HCC) and the overexpression of RASGRP1 was an independent prognostic risk factor in HCC patients [44]. In another study, the interactions between RASGRP1 and the RAS effector kinase CRAF was found to be an important factor that led to drug resistance in lymphoma both in vitro and in vivo [45]. On the contrary, Depeille et al. identified high RASGRP1 expression in colorectal cancer (CRC) patients correlated with a better clinical outcome [46]. Similarly, Wang et al. recently indicated higher expression of RASGRP1 was associated with better DFS and OS for triple-negative breast cancer [47]. Here, we firstly report that the expression level of RASGRP1 mRNA was significantly associated with the prognosis of CSCC. Due to the lack of studies, the molecular mechanisms of RASGRP1 in CSCC remain unclear. Although our results indicate that RASGRP1 may be an intriguing target for CSCC, additional experimental studies should be conducted to support these findings.

To the best of our knowledge, this is the first nomogram for predicting PFS of patients with CSCC that is based on RNA-seq data with long-term follow-up. A comprehensive, easy-to-use scoring system could have a favorable impact on the options of treatment and follow-up schedules for patients with an individualized prediction of PFS probability. Despite our noteworthy findings, this nomogram is limited by the retrospective nature of data acquisition and the failure to integrate some recognized prognostic factors, such as primary tumor size, stromal invasion, and lymphovascular invasion. Further improvements on larger data collecting, incorporation of other prognostic factors, and prospective validation will refine our classifier. Functional analysis of these molecules may provide new insights into mechanisms underlying the progression of CSCC and may help with the discovery of potential therapeutic targets.

In summary, we developed a risk score as defined by an expression pattern of 2 genes for determining the prognosis of CSCC patients. The initial results are promising and a nomogram comprising our prognostic classifier may help predict individual progression risk. The novel co-expression network and machine learning-based strategy described in the study may have a broad application in precision medicine.

## Supplementary information

**Additional file 1: Table S1.** The 8th edition of the International Union Against Cancer (UICC)/American Joint Committee on Cancer (AJCC) Tumor Node Metastasis (TNM) classification and the International Federation of Gynecology and Obstetrics (FIGO) Classifications for Cervical Cancer. **Table S2.** Baseline clinical features for the CSCC patients in the training set and validation set.

**Additional file 2: Figure S1.** The clustering dendrogram of 35 samples and heatmap of clinical traits. **Figure S2.** Determination of soft-thresholding power and dendrogram of all modules. **Figure S3.** Module-trait relationships between module eigengenes and clinical traits. **Figure S4.** Correlations between the gene significance (GS) and module membership (MM) in selected modules. **Figure S5.** Kaplan-Meier analyses of CSCC patients according to the ACAP1 and RASGRP1 status in the training set. **Figure S6.** Kaplan-Meier analyses of CSCC patients according to the ACAP1 and RASGRP1 status in the validation set. **Figure S7.** Volcano plots of differentially expressed genes (DEGs) between high-risk and low-risk groups in the training set and the validation set. **Figure S8.** Comparison of the C-indices of different signatures.

## Data Availability

We declared that materials described in the manuscript, including all relevant raw data, will be freely available to any scientist wishing to use them for non-commercial purposes, without breaching participant confidentiality.

## Abbreviations

CC: Cervical cancer
CSCC: Cervical squamous cell carcinoma
RNA-seq: RNA sequencing
FFPE: Formalin-fixed and paraffin-embedded
WGCNA: Weighted gene co-expression network analysis
LASSO: Least absolute shrinkage and selection operator
FIGO: International Federation of Gynaecology and Obstetrics
PFS: Progression-free survival
OS: Overall survival
HR: Hazard ratio
CI: Confidence interval
HPV: Human papillomavirus
GEO: Gene Expression Omnibus
TCGA: The Cancer Genome Atlas
GDC: Genomic Data Commons
VST: Variance stabilizing transformation
TOM: Topological overlap matrix
MEs: Module eigengenes
GS: Gene significance
MM: With module membership
DEGs: Differential expression genes
C-index: Concordance index
DEA: Gene expression analysis
AUC: Area under the curve
ROC: Receiver operating characteristic curve
LVI: Lymphovascular invasion
GAP: GTPase-activating protein
ARF6: ADP-ribosylation factor 6
ACAP1: ArfGAP with coiled-coil, ankyrin repeat and PH domains 1
RASGRP1: RAS guanyl releasing protein 1
HCC: Hepatocellular carcinoma
CRC: Colorectal cancer
DFS: Disease-free survival
