# Supplementary material for: A prognostic nomogram integrating novel biomarkers identified by machine learning for cervical squamous cell carcinoma
Source: J Transl Med. 2020 Jun 5;18:223. doi: 10.1186/s12967-020-02387-9 (PMC7275455; doi:10.1186/s12967-020-02387-9)
Supplement: Supplementary file 1 — Additional file 1: Table S1. The 8th edition of the International Union Against Cancer (UICC)/American Joint Committee on Cancer (AJCC) Tumor Node Metastasis (TNM) classification and the International Federation of Gynecology and Obstetrics (FIGO) Classifications for Cervical Cancer. Table S2. Baseline clinical features for the CSCC patients in the training set and validation set. [file 12967_2020_2387_MOESM1_ESM.docx]

**A prognostic nomogram integrating novel biomarkers identified by machine learning for cervical squamous cell carcinoma**

Yimin Li^1^, Shun Lu^2,3^, Mei Lan^2^, Xinhao Peng^1^, Zijian Zhang^4^, Jinyi Lang^2,3*^

**Authors Affiliations:**

^1^ School of Medicine, University of Electronic Science and Technology of China, No.2006, Xiyuan Avenue, High-tech Zone (West District), Chengdu City, ZIP 611731, Sichuan Province, People’s Republic of China.

^2^ Department of Radiation Oncology, Sichuan Cancer Hospital & Institute, Sichuan Cancer Center, School of Medicine, University of Electronic Science and Technology of China, No.55, South Renmin Avenue Fourth Section, Chengdu City, ZIP 610041, Sichuan Province, People’s Republic of China.

^3^ Radiation Oncology Key Laboratory of Sichuan Province, No.55, South Renmin Avenue Fourth Section, Chengdu City, ZIP 610041, Sichuan Province, People’s Republic of China.

^4^ Department of Oncology, Xiangya hospital Central South University, Kaifu District, Changsha City, ZIP 410008, Hunan Province, People’s Republic of China.

**^*^Corresponding Authors:** Jinyi Lang, Department of Radiation Oncology, Sichuan Cancer Hospital & Institute, Sichuan Cancer Center, School of Medicine, University of Electronic Science and Technology of China, No.55, South Renmin Avenue Fourth Section, Chengdu City, ZIP 610041, Sichuan Province, People’s Republic of China. Email: langjy610@163.com

**Email addresses:** Yimin Li, ymlee365@126.com; Shun Lu, [lushun1982@live.cn](mailto:lushun1982@live.cn); Mei Lan, merrydoctor@163.com; Xinhao Peng, pengxinhaowangyi@163.com; Zijian Zhang, wanzzj@csu.edu.cn; Jinyi Lang, langjy610@163.com

**Table S1.** The 8th edition of the International Union Against Cancer (UICC) /American Joint Committee on Cancer (AJCC) Tumor Node Metastasis (TNM) classification and the International Federation of Gynecology and Obstetrics (FIGO) Classifications for Cervical Cancer

| TNM Classification | FIGO Stage | Surgical-Pathologic Findings |
| --- | --- | --- |
| Primary tumor (T) |  |  |
| TX |  | Primary tumor cannot be assessed |
| T0 |  | No evidence of primary tumor |
| Tis |  | Carcinoma in situ (preinvasive carcinoma) |
| T1 | I | Cervical carcinoma confined to the cervix (disregard extension to the corpus) |
| T1a | IA | Invasive carcinoma diagnosed only by microscopy; stromal invasion with a maximum depth of < 5.0 mm, measured from the base of the epithelium; vascular space involvement, venous or lymphatic, does not affect classification |
| T1a1 | IA1 | Measured stromal invasion < 3.0 mm in depth |
| T1a2 | IA2 | Measured stromal invasion ≥ 3.0 mm and < 5.0 mm |
| T1b | IB | Invasive carcinoma with measured deepest invasion ≥ 5 mm (greater than stage IA), lesion limited to the cervix |
| T1b1 | IB1 | Invasive carcinoma with ≥ 5 mm depth of stromal invasion and < 2 cm in greatest dimension |
| T1b2 | IB2 | Invasive carcinoma, 2 cm to < 4 cm in greatest dimension |
| T1b3 | IB3 | Invasive carcinoma, ≥ 4 cm in greatest dimension |
| T2 | II | Cervical carcinoma invades beyond uterus but not to pelvic wall or to lower third of vagina |
| T2a | IIA | Involvement limited to the upper two-thirds of the vagina, without parametrial invasion |
| T2a1 | IIA1 | Invasive carcinoma < 4 cm in greatest dimension |
| T2a2 | IIA2 | Invasive carcinoma?≥ 4 cm in greatest dimension |
| T2b | IIB | Tumor with parametrial invasion?but not up to the pelvic wall |
| T3 | III | Carcinoma involves the lower third of the vagina and/or extends to the pelvic wall and/or causes hydronephrosis or nonfunctioning kidney and/or involves pelvic and/or para-aortic lymph nodes |
| T3a | IIIA | Tumor involves lower third of vagina, with no extension to pelvic wall |
| T3b | IIIB | Tumor extends to pelvic wall and/or causes hydronephrosis or nonfunctional kidney |
| T3c | IIIC | Involvement of pelvic and/or para-aortic lymph nodes, irrespective of tumor size and extent (with r [imaging] and p [pathology] notations) |
| T3c1 | IIIC1 | Pelvic lymph node metastasis only |
| T3c2 | IIIC2 | Para-aortic lymph node metastasis |
| T4 | IV | The carcinoma has extended beyond the true pelvis or has involved (biopsy proven) the mucosa of the bladder or rectum. (A bullous edema, as such, does not permit a case to be allotted to stage IV) |
|  | IVA | Spread to adjacent pelvic organs |
|  | IVB | Spread to distant organs |
| Regional lymph nodes (N) |  |  |
| NX |  | Regional lymph nodes cannot be assessed |
| N0 |  | No regional lymph node metastasis |
| N0 (i+) |  | Isolated tumor cells in regional lymph node(s) ≤ 0.2 mm |
| N1 |  | Regional lymph node metastasis |
| Distant metastasis (M) |  |  |
| M0 |  | No distant metastasis |
| M1 |  | Distant metastasis (including peritoneal spread; involvement of supraclavicular, mediastinal, or distant lymph nodes; and lung, liver, or bone) |

**Table S2.** Baseline clinical features for the CSCC patients in the training set and validation set.

| Characteristics | Number of cases（%） | | p-value^1^ |
| --- | --- | --- | --- |
|  | **Training set (n = 36)** | **Validation set (n = 252)** |  |
| Age_Group |  |  | 0.575 |
| <60 | 26(72) | 195(78) |  |
| ≥60 | 10(28) | 55(22) |  |
| Smoking history Category |  |  | 0.018 |
| <3 | 35(97) | 168(79) |  |
| ≥3 | 1(3) | 44(21) |  |
| Histologic_grade |  |  |  |
| 1 |  | 12(5) |  |
| 2 |  | 109(49) |  |
| 3 |  | 102(46) |  |
| 4 |  | 1(0) |  |
| Total Number of Pregnancies |  |  | 0.716 |
| ≤5 | 28(78) | 177(82) |  |
| >5 | 8(22) | 39(18) |  |
| High risk HPV |  |  | 0.051 |
| No | 18(50) | 3(18) |  |
| Yes | 18(50) | 14(82) |  |
| FIGO stage |  |  | < 0.01 |
| I | 0 | 125(51) |  |
| II | 24(67) | 62(25) |  |
| III | 8(22) | 42(17) |  |
| IV | 4(11) | 16(7) |  |
| T stage |  |  | < 0.01 |
| 0 | 0 | 1(0) |  |
| 1 | 0 | 130(53) |  |
| 2 | 24(67) | 68(28) |  |
| 3 | 8(22) | 32(13) |  |
| 4 | 4(11) | 13(5) |  |
| Lymph node status |  |  | 0.083 |
| 0 | 18(50) | 104(67) |  |
| 1 | 18(50) | 51(33) |  |
| Metastasis |  |  | 0.222 |
| 0 | 36(100) | 194(94) |  |
| 1 | 0 | 12(6) |  |
| Lymphovascular invasion (LVI) |  |  |  |
| No |  | 51(44) |  |
| Yes |  | 66(56) |  |
| Radiotherapy |  |  | < 0.01 |
| No | 0 | 105(42) |  |
| Yes | 36(100) | 147(58) |  |
| Chemotherapy |  |  | < 0.01 |
| No | 0 | 136(54) |  |
| Yes | 36(100) | 116(46) |  |
| PFS Status |  |  | 0.086 |
| 0 | 19(54) | 162(70) |  |
| 1 | 16(46) | 68(30) |  |
| Vital status |  |  | 0.551 |
| 0 | 27(82) | 190(75) |  |
| 1 | 6(18) | 62(25) |  |

^1^ p-value is calculated by chi-square test or Fisher exact test.
