# Supplementary material for: A prognostic nomogram integrating novel biomarkers identified by machine learning for cervical squamous cell carcinoma
Source: J Transl Med. 2020 Jun 5;18:223. doi: 10.1186/s12967-020-02387-9 (PMC7275455; doi:10.1186/s12967-020-02387-9)
Supplement: Supplementary file 2 — Additional file 2: Figure S1. The clustering dendrogram of 35 samples and heatmap of clinical traits. Figure S2. Determination of soft-thresholding power and dendrogram of all modules. Figure S3. Module-trait relationships between module eigengenes and clinical traits. Figure S4. Correlations between the gene significance (GS) and module membership (MM) in selected modules. Figure S5. Kaplan-Meier analyses of CSCC patients according to the ACAP1 and RASGRP1 status in the training set. Figure S6. Kaplan-Meier analyses of CSCC patients according to the ACAP1 and RASGRP1 status in the validation set. Figure S7. Volcano plots of differentially expressed genes (DEGs) between high-risk and low-risk groups in the training set and the validation set. Figure S8. Comparison of the C-indices of different signatures. [file 12967_2020_2387_MOESM2_ESM.docx]

**A prognostic nomogram integrating novel biomarkers identified by machine learning for cervical squamous cell carcinoma**

Yimin Li^1^, Shun Lu^2,3^, Mei Lan^2^, Xinhao Peng^1^, Zijian Zhang^4^, Jinyi Lang^2,3*^

**Authors Affiliations:**

^1^ School of Medicine, University of Electronic Science and Technology of China, No.2006, Xiyuan Avenue, High-tech Zone (West District), Chengdu City, ZIP 611731, Sichuan Province, People’s Republic of China.

^2^ Department of Radiation Oncology, Sichuan Cancer Hospital & Institute, Sichuan Cancer Center, School of Medicine, University of Electronic Science and Technology of China, No.55, South Renmin Avenue Fourth Section, Chengdu City, ZIP 610041, Sichuan Province, People’s Republic of China.

^3^ Radiation Oncology Key Laboratory of Sichuan Province, No.55, South Renmin Avenue Fourth Section, Chengdu City, ZIP 610041, Sichuan Province, People’s Republic of China.

^4^ Department of Oncology, Xiangya hospital Central South University, Kaifu District, Changsha City, ZIP 410008, Hunan Province, People’s Republic of China.

**^*^Corresponding Authors:** Jinyi Lang, Department of Radiation Oncology, Sichuan Cancer Hospital & Institute, Sichuan Cancer Center, School of Medicine, University of Electronic Science and Technology of China, No.55, South Renmin Avenue Fourth Section, Chengdu City, ZIP 610041, Sichuan Province, People’s Republic of China. Email: langjy610@163.com

**Email addresses:** Yimin Li, ymlee365@126.com; Shun Lu, [lushun1982@live.cn](mailto:lushun1982@live.cn); Mei Lan, merrydoctor@163.com; Xinhao Peng, pengxinhaowangyi@163.com; Zijian Zhang, wanzzj@csu.edu.cn; Jinyi Lang, langjy610@163.com

**Figure S1：****The clustering dendrogram of 35 samples and heatmap of clinical traits.**


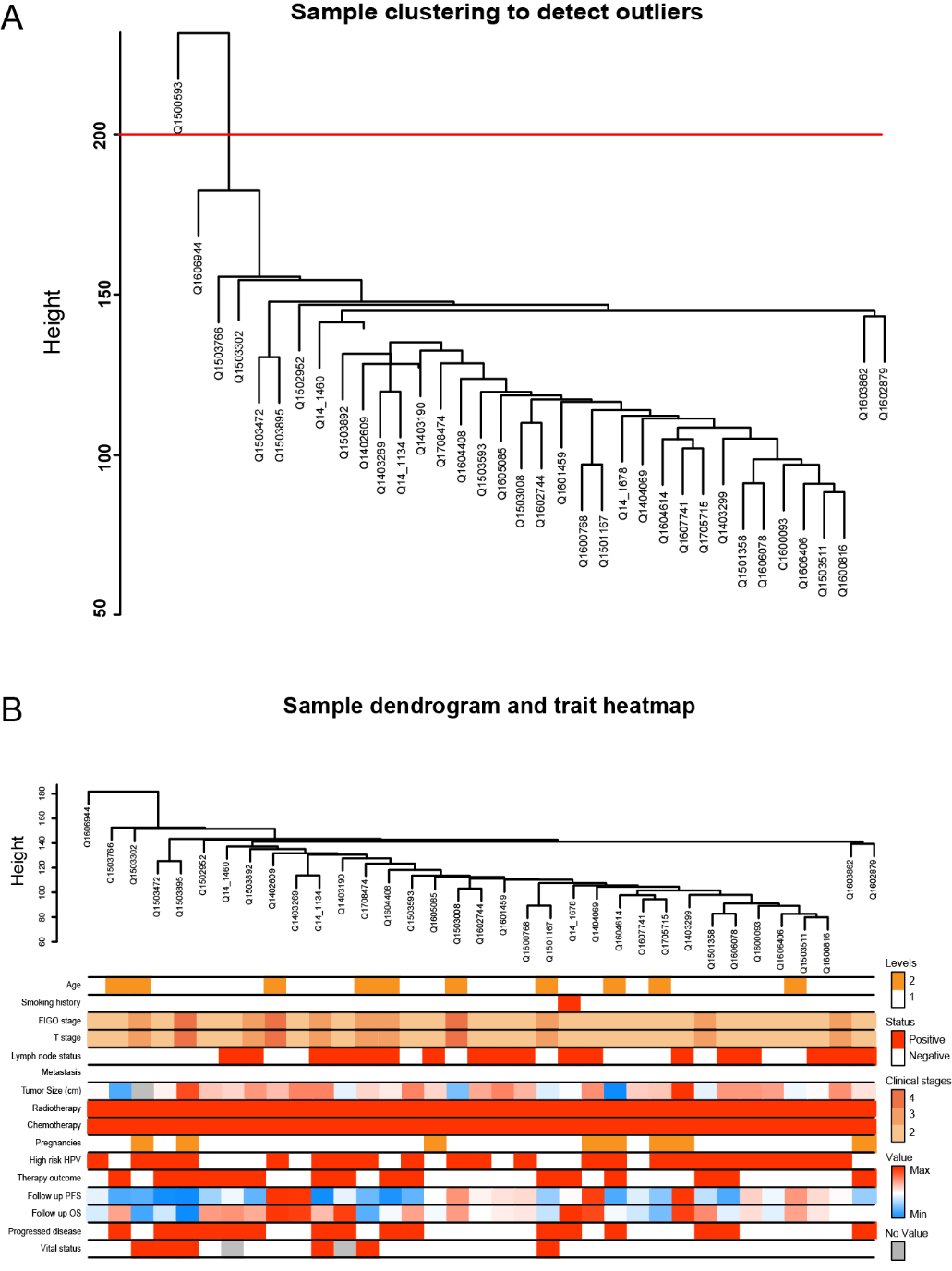


**Figure S1** One sample was deleted as outlier after the hierarchical clustering analysis **(Figure S1A)**. The co-expression network was constructed using 35 cervical squamous cell cancer samples associated with complete clinical data **(Figure S1B)**. For the row of “Age”, level 1 and 2 correspond to <60 years and ≥60 years, respectively. For the “Pregnancies”, level 1 and 2 correspond to ≤5 and >5, respectively.

**Figure S2**：**Determination of soft-thresholding power and dendrogram of all modules.**


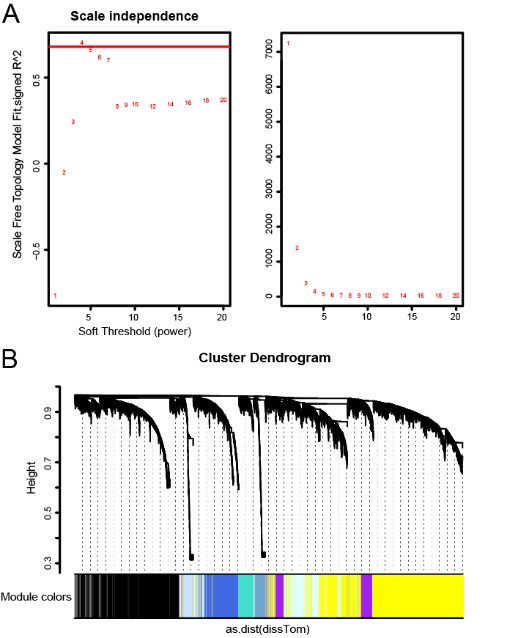


**Figure S2** By the selected power of β = 4 (scale-free R^2^ = 0.703) as the soft-thresholding **(Figure S2A)**, a total of 46 modules were identified **(****Figure S2B)**.

**Figure S3：Module-trait relationships between module eigengenes and clinical traits.**

**
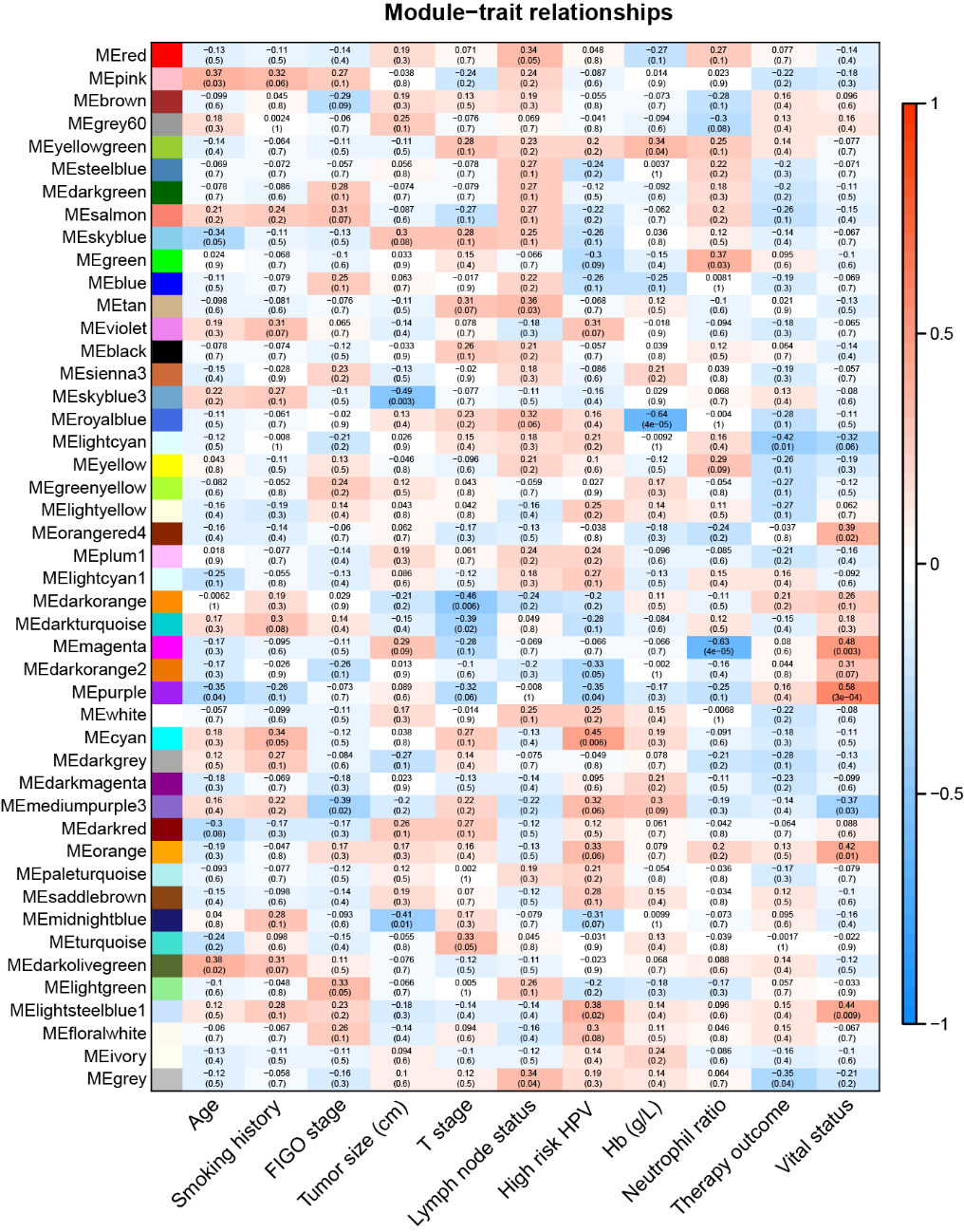
**

**Figure S3** Identification of the correlation between module eigengenes and clinical traits of cervical squamous cell cancer. The corresponding correlation and P-value are at the top and bottom of each cell respectively.

**Figure S4：Correlations between the gene significance (GS) and module membership (MM) in selected modules.**

**
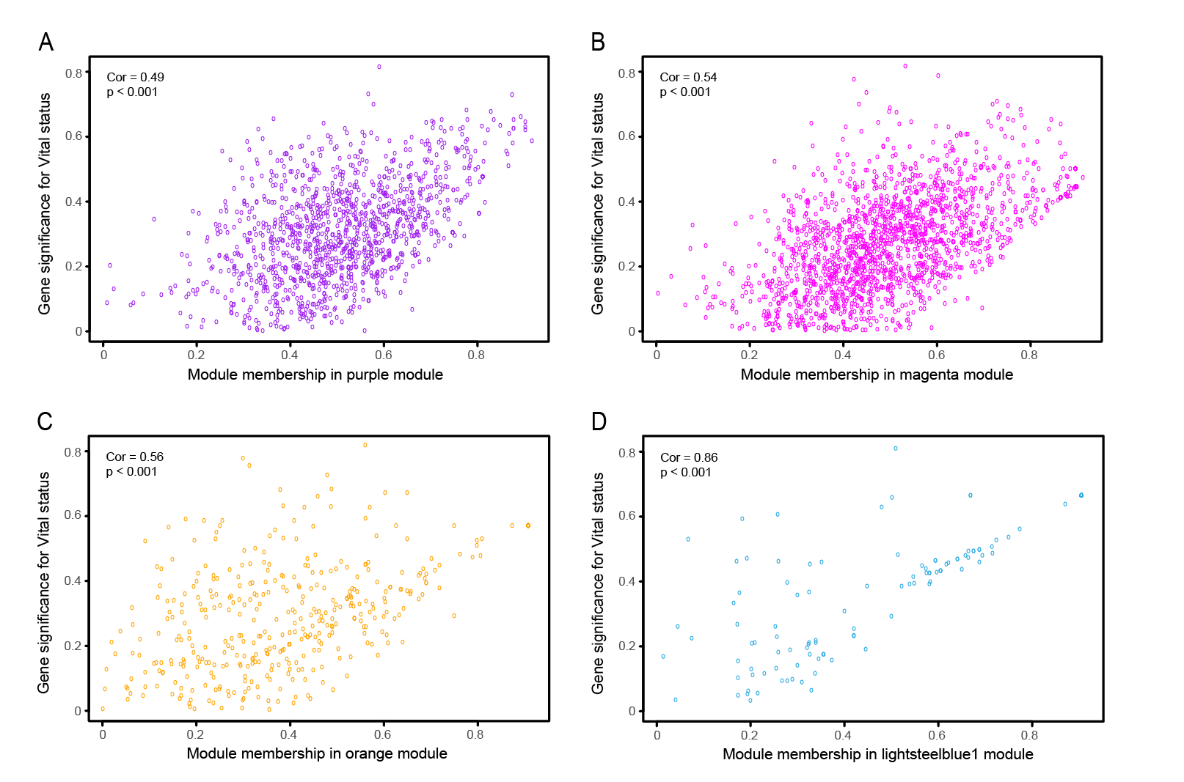
**

**Figure S4** Correlations between the Gene significance (GS) and Module Membership (MM) in the (**Figure S4A**) purple (cor＝0.49, Pearson chi-square test p‐value＜0.001), (**Figure S4B**) magenta (cor＝0.54, Pearson chi-square test p‐value＜0.001), (**Figure S4C**) orange (cor＝0.56, Pearson chi-square test p‐value＜0.001) and (**Figure S4D**) lightsteelblue1 (cor＝0.86, Pearson chi-square test p‐value＜0.001) modules. The y-axis represents the gene significance for the vital status, whereas the x-axis indicates the module membership of genes in each module. Cor represents the absolute correlation coefficient between GS and MM.

**Figure S5：Kaplan-Meier analyses of CSCC patients according to the ACAP1 and RASGRP1 status in the training set.**


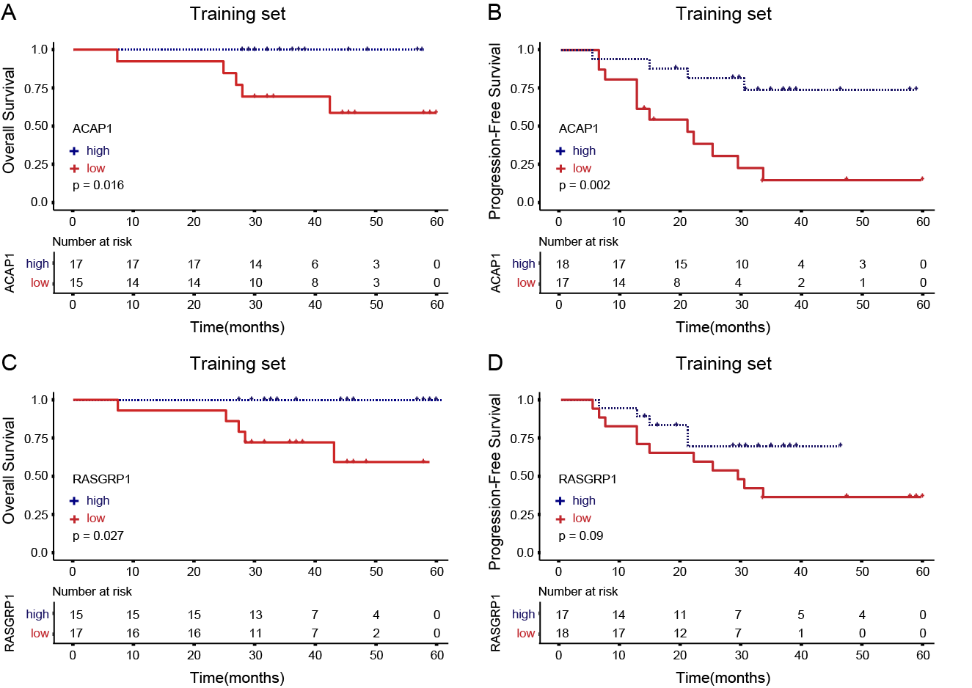


**Figure S5** In the training set, patients with higher ACAP1 showed significantly longer OS (**Figure S5A**, log‐rank test p = 0.016) and PFS (**Figure S5B**, log‐rank test p = 0.002) . RASGRP1 demonstrated the same prognostic value for OS (**Figure S5C**, log‐rank test p = 0.027), but nor for PFS (**Figure S5D**, log‐rank test p = 0.09) .

**Figure S6：Kaplan-Meier analyses of CSCC patients according to the ACAP1 and RASGRP1 status in the validation set.**


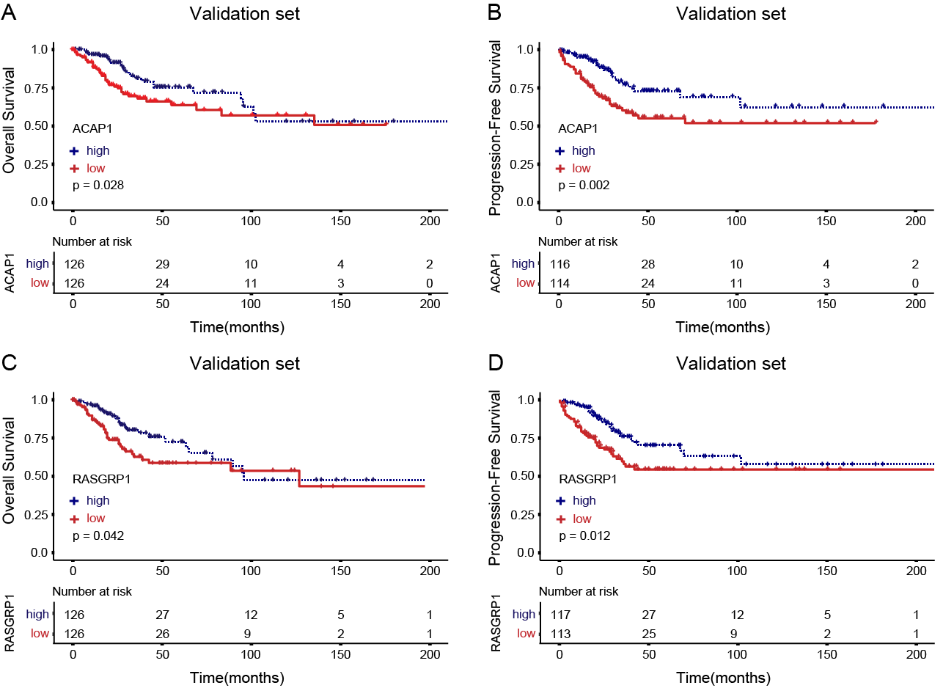


**Figure S6** In the validation set, patients with higher ACAP1 showed significantly longer OS (**Figure S6A**, log‐rank test p = 0.028) and PFS (**Figure S6B**, log‐rank test p = 0.002) . RASGRP1 demonstrated the same prognostic value for OS (**Figure S6C**, log‐rank test p = 0.042) and PFS (**Figure S6D**, log‐rank test p = 0.012) .

**Figure S7：Volcano plots of differentially expressed genes (DEGs) between high-risk and low-risk groups in the training set and the validation set.**


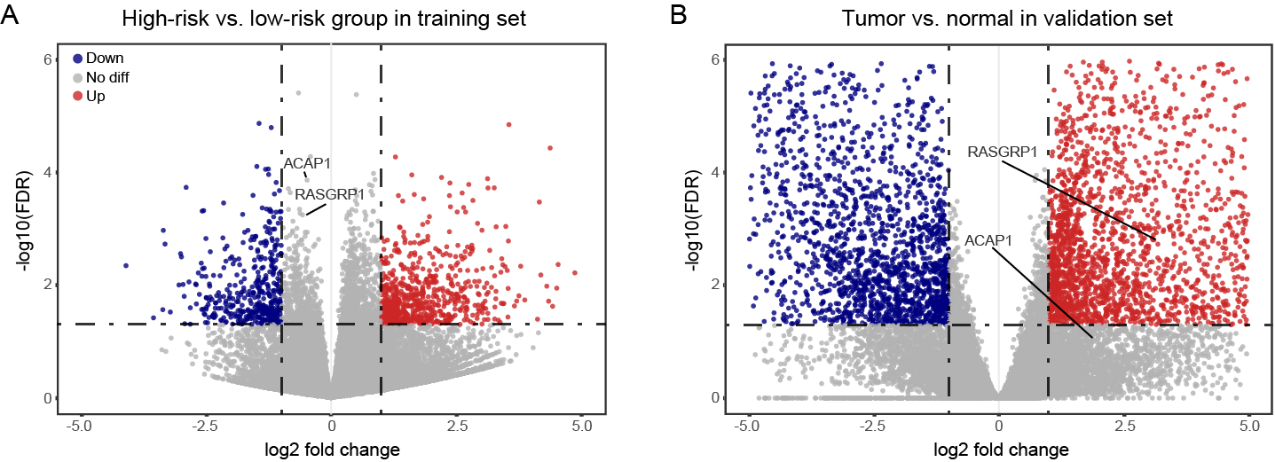


**Figure S7** Volcano plots of differentially expressed genes (DEGs) which were screened by DESeq2 between high-risk and low-risk groups in the training set **(Figure S7A)**; DEGs between tumor and normal samples in the validation set **(Figure S7B)**. Genes with adjusted P-value (false discovery rate, FDR) < 0.05 and |log2 fold change| > 1 are considered as DEGs. The location of ACAP1 and RASGRP1 in volcano plots are indicated by name. The horizontal dashed line indicates the FDR of 0.05. The vertical dashed lines indicate the log2 fold change values of -1 and 1. Blue points: down-regulated genes; Gray points: non-differential genes; Red points: up-regulated genes; FDR, false discovery rate.

**Figure S8**：**Comparison of the C-indices of different signatures.**


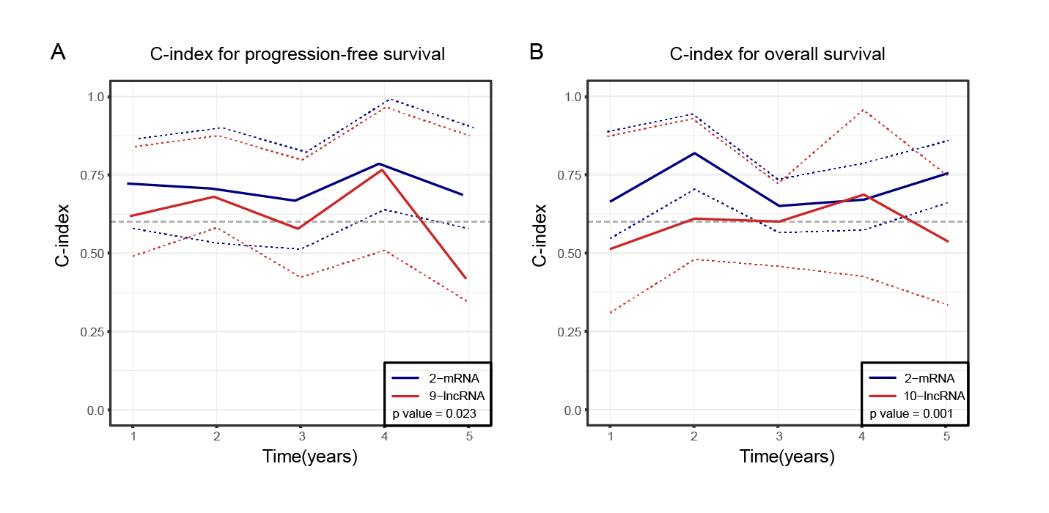


**Figure S8** Comparison of the C-indices of different signatures using the validation set from TCGA. The 2-mRNA based signature had a significantly higher C-index in predicting **(Figure S8A)** PFS (Student t-test p‐value＝0.023) and **(Figure S8B)** OS (Student t-test p‐value＝0.001) at each follow-up duration. The regions between the two dashed lines of the same color provide a 95% confidence interval for the corresponding C-indices. The gray dashed line indicates the C-index value of 0.6.
